# Diffuse cerebellar hypermetabolism: an early sign of leptomeningeal metastases

**DOI:** 10.1007/s00259-021-05358-4

**Published:** 2021-04-17

**Authors:** M. P. G. Broen, M. H. M. E. Anten, J. A. J. van der Pol

**Affiliations:** 1 Department of Neurology, Maastricht University Medical Center+, Mailbox 5800, 6202 AZ Maastricht, The Netherlands; 2 GROW- School for Oncology and Developmental Biology , Maastricht University , Maastricht, The Netherlands; 3 Department of Radiology and Nuclear Medicine, Maastricht University Medical Center+, Maastricht, The Netherlands

A 20-year-old woman was referred to our center with a 3-month history of progressive nausea, headache, and meningismus. She had surgery followed by proton-therapy three years earlier for a right-sided parasagittal atypical meningioma. Extensive blood and CSF examinations were done, but cultures remained negative and no infectious agents or malignant cells were identified. An ^18^F-fluorodeoxyglucose (FDG)-PET/CT showed intense hypermetabolism, exclusively involving the cerebellum, but no other abnormalities (Figure: A/C axial (a) and coronal (c) plane of ^18^F-FDG-PET/CT showing isolated diffuse hypermetabolism of the cerebellum; B/D axial (b) and coronal (d) plane of ^18^F-FDG-PET fused with MR imaging, emphasizing the cortical location of the hypermetabolism). A repeated contrast-enhanced MRI showed new, intense leptomeningeal enhancement of the cerebellar hemispheres, highly suspicious for leptomeningeal metastases. Leptomeningeal biopsy confirmed the diagnosis of leptomeningeal spread of the known meningioma, with rhabdoid transformation (WHO grade 3). Diffuse cerebellar cortical hypermetabolism on ^18^F-FDG-PET/CT is a rare phenomenon reported in a few cases with paraneoplastic cerebellar degeneration [1, 2]. But in this case, paraneoplastic cerebellar antibodies were all negative, in line with the lack of cerebellar signs on examination. Therefore, we postulate a differential diagnosis to this imaging sign, substantiated by pathological proof. Diffuse cerebellar cortical hypermetabolism on ^18^F-FDG-PET/CT can be an early imaging marker for leptomeningeal metastases in solid tumors, appearing even before contrast enhancement on MR imaging. Recognizing this phenomenon may prevent delayed diagnosis and treatment.

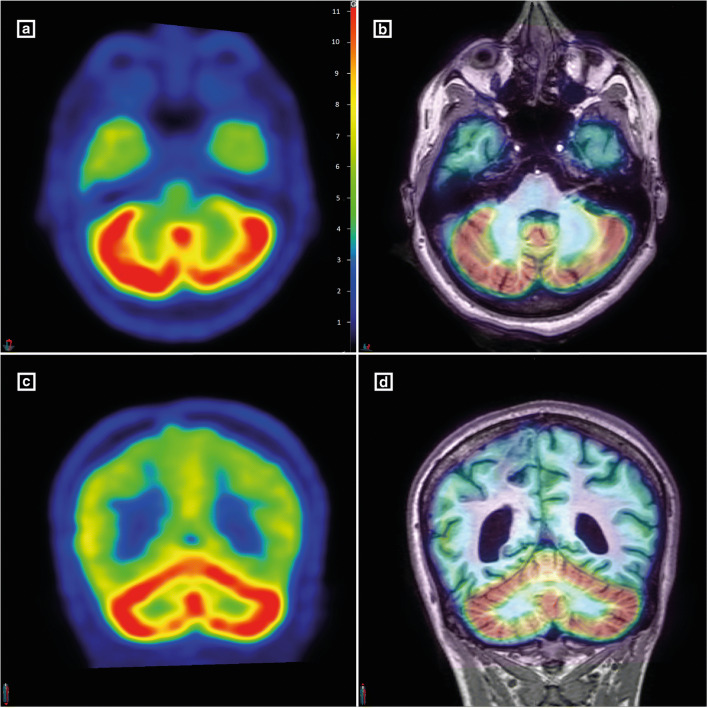



## Data Availability

Not applicable
